# Factors associated with unawareness of HIV-positive status among individuals aged ≥ 15 years in Tanzania: Evidence from the Tanzania HIV Impact Survey 2022–2023

**DOI:** 10.1186/s12889-026-26597-w

**Published:** 2026-03-24

**Authors:** Damian Jeremia Damian, Stephano Cosmas, Alice Wang, Magreth Kagashe, Aisha Haji, Oscar Ernest Rwabiyago, Jocelyn Rwehumbiza, Fahima Issa, Ahmed Khatib, Emilian Karugendo, Samuel Sumba, Rebecca L. Laws, Nicolas Schaad, Mahesh Swaminathan, Sarah E. Porter, Prosper F. Njau

**Affiliations:** 1 Division of Global HIV and TB, U.S. Centers for Disease Control and Prevention, Dar es Salaam, Tanzania; 2 National Bureau of Statistics, Dodoma, Tanzania; 3 Tanzania Commission for AIDS, Dodoma, Tanzania; 4 Office of the Chief Government Statistician, Zanzibar, Tanzania; 5 Zanzibar AIDS Commission, Zanzibar, Tanzania; 6 Division of Global HIV and TB, U.S. Centers for Disease Control and Prevention, Atlanta, GA USA; 7 National AIDS, STI, and Hepatitis Control Programme, Dodoma, Tanzania

**Keywords:** HIV-testing, HIV-infections, Condoms, Awareness, Tanzania

## Abstract

**Introduction:**

In Tanzania, despite the progress in HIV testing and counseling, 39.4% of people living with HIV aged ≥ 15 years were unaware of their HIV status in 2017. HIV testing is a crucial entry point for prevention, early diagnosis, and treatment of HIV to increase survival, improve quality of life, and prevent further transmission. This analysis aimed to estimate the proportion of individuals aged ≥ 15 years living with HIV in Tanzania who were unaware of their HIV status and to determine factors associated with unawareness of HIV-positive status.

**Methods:**

We analyzed data from the Tanzania HIV Impact Survey 2022–2023, a nationally representative cross-sectional survey which employed a two-stage cluster sampling design and included HIV testing at the household with laboratory confirmation. This analysis focused on individuals aged ≥ 15 years who tested HIV-positive during the survey. Weighted percentages were calculated to summarize demographic information and the proportion unaware of their HIV-positive status. Logistic regression accounting for complex survey design was used to determine factors associated with unawareness of HIV-positive status.

**Results:**

A total of 1,850 individuals aged ≥ 15 years tested HIV positive, among which 266 (17.3%) were unaware of their HIV-positive status. Males were more likely to be unaware of their HIV-positive status than females (21.6%, aOR = 1.67, 95% CI: 1.06–2.61, *p* = 0.029 vs. 15.2%). Young people aged 15 − 24 years were over five times more likely to be unaware of their HIV-positive status (aOR = 5.61, 95% CI: 2.12–14.84, *p* = 0.001) compared to individuals aged ≥ 55 years. Individuals who reported not using a condom at last sexual intercourse in the past year were more likely to be unaware of their HIV status than those who reported condom use (aOR = 2.36, 95% CI: 1.32–4.20, *p* = 0.005). Those with at least one partner living with HIV were less likely to be unaware of their HIV-positive status compared to individuals with all sexual partners who were HIV negative (aOR = 0.04, 95% CI: 0.00-0.42, *p* = 0.009).

**Conclusion:**

Despite robust HIV case-finding interventions in Tanzania, 1 in 6 PLHIV were unaware of their HIV-positive status. Targeted HIV case-finding interventions should continue with a focus on reaching men and young people who were more likely to be unaware of their status. HIV prevention strategies, such as condom use, remain crucial, since individual behaviors were linked to awareness of HIV status.

## Introduction

In 2014, the Joint United Nations Programme on HIV/AIDS (UNAIDS) launched the Fast-Track strategy for ending HIV/AIDS as a public health threat by the year 2030 [1]. The strategy focuses strongly on the 30 countries that account for 89% of new HIV infections globally, including the United Republic of Tanzania [2]. Fast-Track targets include reducing the number of adults newly acquiring HIV infection globally from 2.1 million in 2010 to fewer than 200,000 in 2030; averting 28 million HIV infections in low- and middle-income countries between 2015 and 2030; and averting 21 million AIDS-related deaths. The Fast-Track target for HIV treatment provides that, by 2025, 95% of people living with HIV (PLHIV) should be aware of their HIV status (the first 95); 95% of those who know their HIV-positive status should be on antiretroviral treatment (ART) (the second 95); and 95% of those who are on treatment should have a suppressed viral load (VL) (the third 95) [1].

HIV testing services are the entry point for identifying PLHIV and linking them to life-saving ART [3]. Achieving the first 95 target for awareness of HIV-positive status is also an essential first step for preventing further HIV transmission and ultimately controlling the HIV epidemic. Findings from the Tanzania HIV Impact Survey 2016–2017 (THIS 2016–2017) illustrated the critical importance of the first 95. The survey found that only 60.6% of individuals aged ≥ 15 years living with HIV in Tanzania were aware of their HIV-positive status [4]. Although 93.6% of those who knew their status were on ART and 87.0% of those on ART had suppressed VL, the large gap in awareness, the first 95, meant that only 56.7% of PLHIV aged ≥ 15 years in Tanzania were on ART [4].

In the THIS 2016–2017, higher proportions of men (54.8%), young people aged 15–24 years (60.9%), rural residents (52.9%), and those in the lowest two wealth quintiles (61.6%) were unaware of their HIV-positive status. For men living with HIV, unawareness of HIV status was higher among those who were never married (66.7%) or divorced or separated (64.3%) compared to those who were married or cohabiting (53.5%). For women living with HIV, unawareness of HIV status was higher among those with no education (54.3%) compared to those with at least primary education (41.7%) [4].

Many studies in sub-Saharan Africa have found associations between unawareness of HIV-positive status and socio-demographic variables. Men and young people aged 15–24 years are generally less likely to know their HIV status [5]. Analyses of population-based HIV impact assessment (PHIA) data from multiple countries have also confirmed associations between younger age and unawareness of HIV-positive status in sub-Saharan Africa [6–9]. Additionally, multiple studies have consistently found lower awareness of HIV status among those who were less educated, never married, or coming from households headed by people who were not living with HIV [6, 10–12]. In Tanzania, lower wealth, living in rural areas, and multiple sex partners are also factors that have been shown to be associated with unawareness of HIV-positive status [7].

We estimated the proportion of individuals ≥ 15 years living with HIV who were unaware of their HIV status in Tanzania and determined factors associated with unawareness of HIV status using THIS 2022–2023 data. This analysis will help guide the Government of Tanzania (GOT) and partners to understand the extent of current gaps in knowledge of HIV status, target interventions to reach specific sub-populations with HIV testing services, and decrease disparities in HIV testing coverage, especially among sub-populations with higher rates of HIV incidence.

## Methods

### Study design and participants

We analyzed data from the THIS 2022–2023, a nationally representative cross-sectional survey conducted between November 2022-March 2023 which employed a two-stage cluster sampling design. The first stage involved the selection of enumeration areas (EAs) using probability proportional to size from the sampling frame of the 2022 Population and Housing Census, stratified by geographical region across the 31 regions of Tanzania [13]. In total, 566 EAs were selected in the first stage. In the second stage, an average of 35 households were selected from each of the selected EAs. Individuals aged ≥ 15 years who slept in selected households the previous night were eligible to give consent to participate in individual interviews and HIV testing. This analysis focused on individuals aged ≥ 15 years who tested positive for HIV during the survey. The methodology of the THIS 2022–2023 has been previously described elsewhere [14, 15].

### Data collection

Self-reported awareness of HIV-positive status, along with demographic, behavioral, and clinical data, was collected through interviewer-administered questionnaires. HIV testing was conducted using Tanzania’s national HIV testing algorithm [16]. HIV test results were returned to the participants on the same day. Laboratory confirmation of seropositive samples was done using a BioRad GeeniusTM HIV-1/HIV-2 supplemental assay. Qualitative screening for a detectable concentration of antiretroviral (ARV) drugs was conducted on dried blood samples of all HIV-positive samples by high-resolution liquid chromatography coupled with tandem mass spectrometry, a modified version of methodology described by Koal et al. [17]. ARVs tested for included dolutegravir, efavirenz, atazanavir, and lopinavir, which are the most prescribed first- and second- line regimes in Tanzania.

### Variable definition

**Fig. 1:**
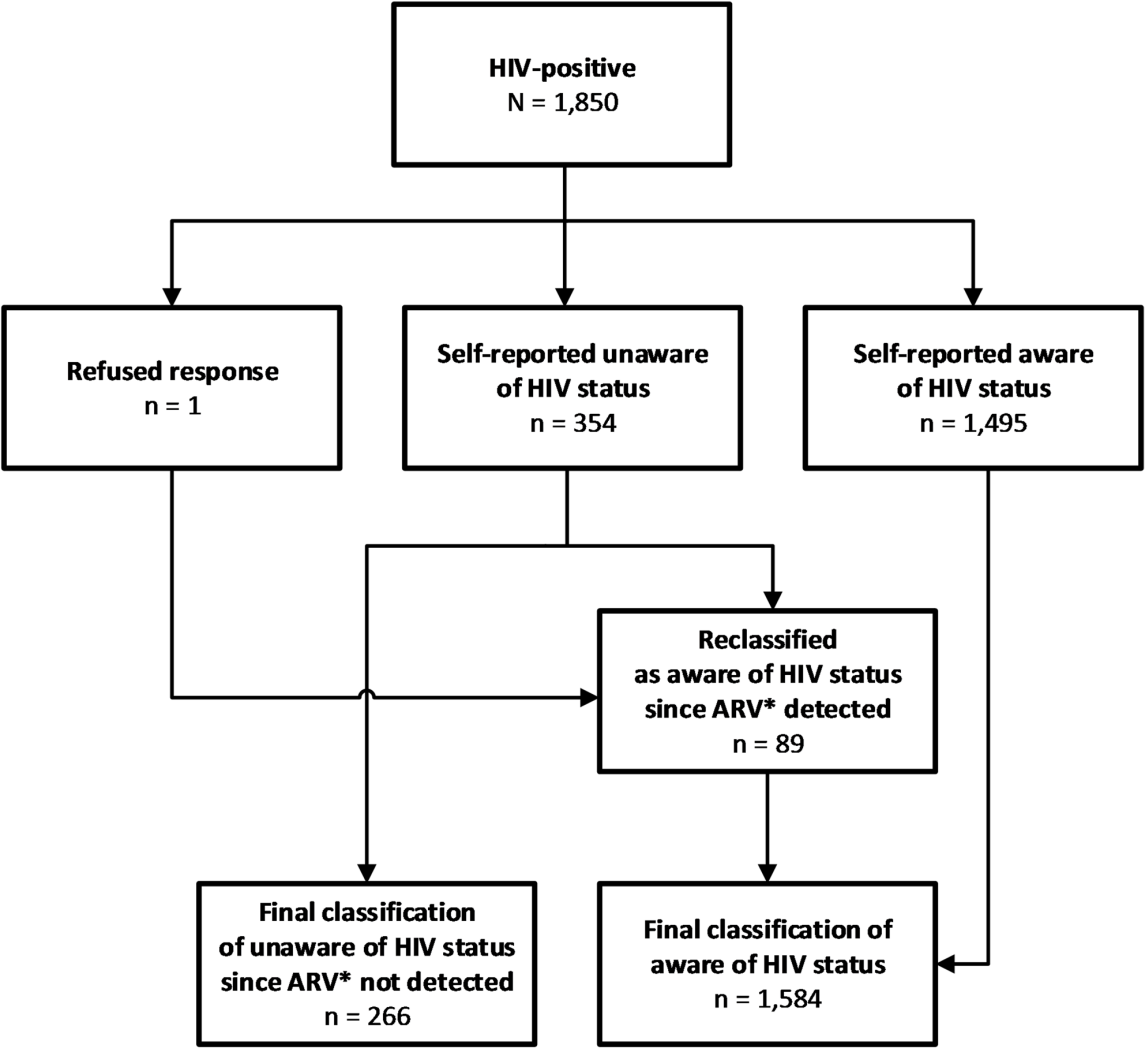
Distribution of individuals living with HIV aged ≥ 15 years by HIV status awareness in Tanzania, Tanzania HIV Impact Survey 2022–2023 (*N* = 1,850). *ARV: Antiretroviral

The primary outcome variable used in our analysis was unawareness of HIV-positive status, described as a binary outcome: aware or unaware. Participants were classified as aware of their HIV-positive status if they reported knowing they were HIV-positive before survey-related testing or if they had an ARV detected in their blood (Fig. 1). The explanatory variables included demographic variables such as sex, age, residence, marital status, education level and wealth. Wealth quintiles were dichotomized into two categories: lower 40% and upper 60%. We also included behavioral characteristics such as ever had sexual intercourse, first sex before aged 15 years, number of sexual partners in the past 12 months, used condom during the last sexual intercourse in the past 12 months, time since last HIV test defined as number of years elapsed since participants received HIV test from the interview date, partners’ HIV status, and alcohol use defined as hazardous drinking or non-hazardous drinking, determined by the World Health Organization’s “Alcohol Use Disorders Identification Test – Concise” (AUDIT-C) [18]. Partners’ HIV status was defined for sexual partnerships in the past 12 months and included 4 categories assigned sequentially: negative if thought, told, or tested together as negative for all of their partners; positive if thought, told, or tested together as positive for one or more of their partners; unknown status for one or more of their partners; and no sexual partners.

### Statistical analysis 

Data were analyzed using Stata Software version 18 (StataCorp. 2023. Stata Statistical Software: Release 18. College Station, TX: StataCorp LLC). Categorical variables were summarized using frequencies and proportions. Descriptive analyses were conducted to describe the population characteristics, and unawareness of HIV-positive status. Jackknife variance estimation was used to generate 95% confidence intervals (CI). Logistic regression analysis was used to determine associations between participants’ unawareness of their HIV positive status and the potential explanatory variables. Variables that were found to be significant at the 10% level in the bivariate analysis were included in the multivariate model. Multicollinearity was assessed using variance inflation factors (VIFs), model fit was evaluated using the Hosmer–Lemeshow goodness-of-fit test and McFadden’s pseudo R2. A *p*-value < 0.05 was considered statistically significant in all tests. Analyses were weighted for non-response and accounted for the survey design.

## Results

### Characteristics of people living with HIV

A total of 1,850 individuals aged ≥ 15 years who tested positive for HIV were included in the analysis. The mean age of PLHIV was 44 (SD ± 12.8) years, 66.8% were female, 45.7% were aged 35 − 54 years, 56.1% lived in rural areas, 68.3% had primary school as the highest level of school attended, 51.7% were married or living together, 98.5% reported ever having sexual intercourse, and 8.5% reported having first sex before aged 15 years. Among those who reported ever having sexual intercourse, 18.5% reported having had multiple sexual partners in the past 12 months, and 76.8% reported not using a condom during at last sexual intercourse in the past 12 months. Among PLHIV, 39.5% were tested for HIV in the past 12 months, 31.1% reported having at least one sexual partner with unknown HIV status in the past 12 months, and 17.6% reported hazardous drinking (Table 1).

**Table 1:** Characteristics and prevalence of unawareness of HIV-positive status among individuals living with HIV aged ≥ 15 years in Tanzania, Tanzania HIV Impact Survey 2022–2023

Variables	*n* (%)	% Unaware of HIV-positive status (95% CI)
Sex (*N* = 1,850)		
Male	551 (33.2)	21.6 (17.2 − 26.9)
Female	1,299 (66.8)	15.2 (12.8 − 17.9)
Residence		
Urban	681 (43.9)	17.8 (14.0 − 22.3)
Rural	1,169 (56.1)	17.0 (14.0 − 20.4)
Age (years) (*N* = 1,850)		
15 − 24	115 (7.4)	32.3 (22.1 − 44.7)
25 − 34	339 (18.9)	19.1 (14.6 − 24.6)
35 − 44	525 (29.0)	19.0 (14.6 − 24.2)
45 − 54	513 (16.7)	14.0 (10.3 − 18.8)
≥ 55	358 (18.0)	11.6 (8.16 − 16.3)
Highest level of school attended (*N* = 1,847)		
None	312 (16.6)	21.0 (15.0 − 28.6)
Primary	1,290 (68.3)	15.9 (13.4 − 18.9)
Secondary and above	245 (15.1)	20.0 (14.2 − 27.3)
Marital status (*N* = 1,848)		
Never married	191 (12.4)	24.3 (17.8 − 32.4)
Married or living together	963 (51.7)	18.9 (15.7 − 22.5)
Divorced or separated	372 (20.0)	13.7 (9.8 − 18.6)
Widowed	322 (15.9)	11.6 (8.0 − 16.5)
Wealth category (*N* = 1,850)		
Lower 40%	789 (41.8)	17.4 (13.4 − 22.2)
Upper 60%	1,061 (58.2)	17.3 (14.3 − 20.8)
Ever had sexual intercourse (*N* = 1,842)		
Yes	1,823 (98.5)	17.4 (15.1 − 20.1)
No	19 (1.5)	18.9 (5.7 − 47.6)
Had first sex before aged 15 years (*N* = 1,717)		
Yes	134 (8.5)	22.4 (14.1 − 33.6)
No	1,564 (91.5)	17.9 (15.4 − 20.6)
Number of sexual partners in past 12 months (*N* = 1,739)		
0	402 (21.9)	12.9 (9.5–17.4)
1	1,099 (81.5)	18.8 (15.6 − 22.4)
≥ 2	238 (18.5)	21.6 (16.0 − 28.4)
Used condom at last sexual intercourse in past 12 months (*N* = 1,736)		
Yes	295 (23.2)	11.8 (7.9 − 17.2)
No	1,039 (76.8)	21.6 (18.2 − 25.5)
No sexual intercourse	402 (21.1)	12.9 (9.5–17.4)
Tested for HIV in the past 12 months (*N* = 1,776)		
Yes	673 (39.5)	15.0 (11.4 − 19.6)
No	1,103 (60.5)	18.5 (15.1 − 22.4)
Time since last HIV test (years) (*N* = 1,730)		
≤ 1	809 (47.7)	15.0 (11.7 − 19.0)
2 − 4	345 (19.8)	21.0 (16.0 − 27.1)
5 − 9	305 (17.5)	14.5 (10.1 − 20.4)
≥ 10	271 (15.0)	2.3 (0.9 − 6.1)
Partners’ HIV status in past 12 months (*N* = 1,758)		
All partners negative for HIV	387 (23.3)	24.2 (18.8–30.5)
At least one partner living with HIV	466 (23.7)	1.2 (0.3–5.1)
At least one partner with unknown HIV status	484 (30.7)	29.6 (24.5–35.2)
No sexual partners	421 (22.3)	13.3 (9.9–17.7)
Alcohol use (*N* = 1,771)		
Non-hazardous drinking	1,407 (82.4)	15.3 (11.0 − 20.8)
Hazardous drinking	364 (17.6)	17.5 (15.0 − 20.4)

### Unawareness of HIV-positive status

Of the 1,850 individuals aged ≥ 15 years who tested positive for HIV, 23.1% self-reported being unaware of their HIV-positive status. Among those who reported being unaware of their HIV-positive status, 24.9% had detectable ARVs in their blood sample and were thus classified as being aware of their HIV-positive status. For our analysis, 266 PLHIV (17.3%) were classified as being unaware of their HIV-positive status and 1,584 PLHIV (82.7%) were classified as aware (Fig. 1).

Unawareness of HIV-positive status was higher among men (21.6%, 95% CI: 17.2 − 26.9) than among women (15.2%, 95% CI: 12.8 − 17.9) (Table 1). Unawareness of HIV-positive status was comparable across urban (17.8%, 95% CI: 14.0 − 22.3) and rural residence (17.0%, 95% CI: 14.0 − 20.4), as well as across the lower 40% of wealth (17.4%, 95% CI: 13.4 − 22.2) and upper 60% of wealth (17.3%, 95% CI: 14.3 − 20.8). Unawareness among young people aged 15 − 24 years (32.3%, 95% CI: 22.1 − 44.7) was higher than among older individuals and unawareness decreased with age. Unawareness of HIV-positive status was also higher among those who reported no condom use at last sexual intercourse in the past 12 months (21.6%, 95% CI: 18.2 − 25.5) compared to those who reported condom use (11.8%, 95% CI: 7.9 − 17.2) (Table 1). Unawareness of HIV-positive status was lowest (1.2%, 95% CI: 0.3–5.1) among individuals with at least one partner living with HIV.

### Factors associated with unawareness of HIV-positive status

Sex, age, no condom use at last sexual intercourse in the past 12 months, time since last HIV test, and partners’ HIV status in the past 12 months were independently associated with unawareness of HIV-positive status in the multivariate logistic regression analysis. Males were more likely to be unaware of their HIV-positive status than females.

(aOR = 1.67, 95% CI: 1.06–2.61, *p* = 0.029). Individuals aged 15–24 years (aOR = 5.61, 95% CI: 2.12–14.84, *p* = 0.001); 25–34 years (aOR = 2.44, 95% CI: 1.09–5.48, *p* = 0.032); 35–44 years (aOR = 2.80, 95% CI: 1.27–6.17, *p* = 0.013); and 45–54 years (aOR = 2.30, 95% CI: 1.06–4.98, *p* = 0.035) were more likely to be unaware of their HIV-positive status compared to individuals aged ≥ 55 years. Individuals who reported not using a condom at last sexual intercourse in the past 12 months were more than twice as likely to be unaware of their HIV status than those who reported condom use (aOR = 2.36, 95% CI: 1.32–4.20, *p* = 0.005). Compared to individuals who had tested for HIV test in the past 12 months, individuals who had tested for HIV ≥ 10 years ago were less likely to be unaware of their HIV-positive status (aOR = 0.19, 95% CI: 0.06–0.57, *p* = 0.005). Individuals with at least one partner living with HIV were less likely to be unaware of their HIV-positive status (aOR = 0.04, 95% CI: 0.00-0.42, *p* = 0.009) compared to individuals with all sexual partners who were HIV negative (Table 2).

**Table 2:** Factors associated with unawareness of HIV-positive status among individuals living with HIV aged ≥ 15 years in Tanzania, Tanzania HIV Impact Survey 2022–2023^*^

Variables	Crude			Adjusted			
	cOR (95%CI)	*p*-value		cOR (95%CI)		*p*-value	
Sex (*N* = 1,850)							
Female	1			1			
Male	1.54 (1.12 − 2.1)	0.009		1.66 (1.06–2.61)		0.029	
Residence (*N* = 1,850)							
Urban	1						
Rural	0.95 (0.65 − 1.3)	0.759					
Age (years) (*N* = 1,850)							
15–24	3.63 (2.01 − 6.5)	< 0.001		5.61 (2.12–14.84)		0.001	
25–34	1.79 (1.10 − 2.9)	0.022		2.44 (1.09–5.48)		0.032	
35–44	1.76 (1.07 − 2.9)	0.028		2.80 (1.27–6.17)		0.013	
45–54	1.23 (0.76 − 2.0)	0.376		2.30 (1.06–4.98)		0.035	
≥ 55	1			1			
Highest level of school attended (*N* = 1,847)							
None	1						
Primary	0.71 (0.46 − 1.0)	0.115					
Secondary and above	0.94 (0.54 − 1.6)	0.811					
Marital Status (*N* = 1,848)							
Never married	1			1			
Married or living together	0.72 (0.47 − 1.1)	0.134		1.05 (0.56–1.99)		0.867	
Divorced or separated	0.49 (0.29 − 0.8)	0.009		0.74 (0.38–1.43)		0.351	
Widowed	0.41 (0.23 − 0.7)	0.003		0.76 (0.28–2.05)		0.578	
Wealth category (*N* = 1,850)							
Lower 40%	1						
Upper 60%	0.99 (0.67 − 1.4)	0.980					
Ever had sexual intercourse (*N* = 1,842)							
Yes	0.90 (0.21 − 3.8)	0.887					
No	1						
Had first sex before aged 15 years (*N* = 1,717)							
Yes	1						
No	0.76 (0.43 − 1.3)	0.317					
Number of sexual partners in past 12 months (*N* = 1,739)							
0	1						
1	1.70 (1.12–2.58)	0.014					
≥ 2	2.03 (1.23–3.32)	0.007					
Used condom at last sexual intercourse in past 12 months (*N* = 1,736)							
Yes	1			1			
No	2.07 (1.27–3.38)	0.005		2.36 (1.32–4.20)		0.005	
No sexual intercourse	1.02 (0.58–1.79)	0.938		0.93 (0.43-2.00)		0.837	
Tested for HIV in the past 12 months (*N* = 1,776)							
Yes	1						
No	1.28 (0.84 − 1.9)	0.236					
Time since last HIV test (years) (*N* = 1,730)							
≤ 1	1			1			
2 − 4	1.51 (0.97 − 2.3)	0.068		1.54 (0.95–2.52)		0.076	
5 − 9	0.96 (0.56 − 1.6)	0.881		1.27 (0.76–2.11)		0.342	
≥ 10	0.13 (0.05 − 0.4)	0.001		0.19 (0.06–0.57)		0.005	
Partners’ HIV status in past 12 months (*N* = 1,758)							
All partners negative for HIV	1			1			
At least one partner living with HIV	0.04 (0.00-0.39)	0.008		0.04 (0.00-0.42)		0.009	
At least one partner with unknown HIV status	1.32 (0.85–2.02)	0.201		1.10 (0.67–1.82)		0.696	
No sexual partner	0.48 (0.32–0.73)	0.001		-			
Alcohol use (*N* = 1,1771)							
Non-hazardous drinking	1						
Hazardous drinking	0.85 (0.58 − 1.05)	0.397					

*cOR *crude odds ratio, *aOR *adjusted odds ratio

^*^Statistical diagnostic tests supported the adequacy of the final logistic regression model: variance inflation factor of < 2.34, Hosmer-Lemeshow goodness-of-fit with *p* = 0.893, and McFadden’s *R*^2^ goodness-of-fit of 19% (*p* < 0.001)

## Discussion

Results from the THIS 2022–2023 demonstrated significant progress towards the achievement of the first 95, awareness, among individuals aged ≥ 15 years in Tanzania compared with the THIS 2016 − 2017. The proportion of PLHIV who were unaware of their HIV-positive status decreased by more than half, from 39.4% to 17.3% [4]. The improvement might be attributed to a number of policies and programmatic interventions that were pursued between the two surveys. First, lowering of the age of consent for HIV testing in Tanzania from aged 18 years to 15 years removed a major barrier to reaching younger people with testing services [19]. Additionally, a number of targeted and evidence-based case-finding interventions were introduced and scaled by the GOT in response to THIS 2016 − 2017 findings [20, 21]. For example, from 2018 onwards, the implementation of index testing (identifying and testing the HIV-exposed contacts of a PLHIV) was strengthened and scaled and has been shown to be one of most effective HIV case finding modalities in Tanzania, accounting for 13% of total new HIV positive cases identified in 2018 to over 50% from 2020 onwards [22, 23]. In another example, HIV self-testing services for hard-to-reach populations were introduced in Tanzania in 2018 and scaled across the country by 2020 [20, 21]. More recently in 2021, the social network strategy (identifying and testing social networks of an individual at high-risk) for HIV testing was also introduced [23]. Nonetheless, despite these efforts, one in six PLHIV in Tanzania still were unaware of their HIV status and disparities in testing coverage persist.

We found HIV-positive males and younger people (aged 15–24 years) to be significantly less likely to be aware of their HIV-positive status. These findings are in accordance with numerous studies in sub-Saharan Africa that have shown lower awareness of HIV status among men and underline the importance of sustained, male-focused HIV testing interventions and continued innovation to identify and engage hard-to-reach men [5, 10, 11, 24, 25]. Our findings are also supported by other studies that have found lower HIV testing coverage and lower awareness of HIV-positive status among adolescents and young people (AYP) in sub-Saharan Africa [7, 26–28]. Lower awareness of HIV-positive status among both men and AYP might be explained by their lower uptake of healthcare services compared with women and older individuals [10]. Furthermore, AYP might be dissuaded from accessing HIV testing services due to fears of HIV-related stigma and judgement for risky sexual behaviors [7, 26]. Following the initial release of results from the THIS 2022–2023, the GOT, made programmatic shifts to address the gap among AYP and men. Some of the AYP and men-friendly services instituted include leveraging digital technology for HIV testing demand creation, universal testing for AYP at health facility testing points, remapping of youth and male hangouts for targeted community interventions, and the use of AYP and male peer providers for HIV testing services. In addition, targeted community testing was enhanced in areas commonly frequented by men such as fishing communities, parking areas utilized by long-distance truck drivers, pick-up/drop off areas utilized by motorbike taxi drivers (known in East Africa as “*bodaboda*”) and mines [29]. Moving forward, it will be critical to monitor the impact of these programmatic interventions on awareness in these important groups.

We also found that PLHIV who reported not using condoms during their last sexual encounter were over two and half times more likely to be unaware of their status than those who reported using a condom. Studies in South Africa have also found that PLHIV who are unaware of their status are less likely to use condoms consistently than those who are aware [7, 28]. Since our results come from a cross-sectional survey, we cannot establish the causal relationship between HIV-positive awareness and condom use. However, these results highlight an important association between a key HIV prevention behavior and awareness of status, whereby individuals might be less likely to engage in condom use if they do not know that they are HIV-positive. This underlines the importance of concurrent implementation of HIV case-finding prevention activities.

We did not find having multiple sex partners to be significantly associated with unawareness of HIV-positive status. This is consistent with findings reported elsewhere [19]. Several factors might explain this. At the time of the THIS 2022–2023, the implementation of index testing in Tanzania had been fully scaled for over 5 years and sexual partners of known PLHIV were consistently elicited and tested at high rates. In addition, having multiple sexual partners is one of the risk factors assessed in the national HIV risk screening process which is administered to all individuals attending health facilities in order to identify those to be tested for HIV. The increasingly widespread availability of HIV self-testing might also facilitate testing through the secondary distribution of test kits to sexual partners and targeted community testing focused on hot spots frequented by individuals who engage in commercial sex work.

In addition, we did not observe any significant differences in unawareness of HIV-positive status by education level, marital status, or place of residence (urban versus rural). The widespread availability of HIV testing services across health facilities throughout Tanzania, along with community outreach efforts, might help explain the lack of variation across these factors. Although these characteristics have been associated with unawareness of HIV-positive status in some settings, findings have not been consistent across countries [7].

The THIS 2022–2023 data are subject to several limitations. Despite adjusting for the presence of ARVs in blood samples when estimating awareness of HIV-positive status, participants who were aware of their status but did not disclose it might still have been misclassified as unaware, resulting in an underestimation of awareness. The detection of ARVs in blood samples was limited to publicly-used treatment regimens in Tanzania in 2022–2023, and any PLHIV using other, uncommon regimens would have been classified as not having detectable ARVs in their blood samples. Additionally, there are limitations related to recall bias and reporting bias that might have affected the reported responses and thus our reported associations. Since this analysis was restricted to individuals aged ≥ 15 years, another significant limitation is the lack of data for children where important case finding gaps remain, although risk factors might differ.

## Conclusion

Despite the tremendous progress that the GOT has made in case-finding since the THIS 2016–2017, one in six PLHIV were still unaware of their HIV-positive status in Tanzania. Results from this analysis highlight the need for continued efforts to accelerate progress towards awareness, the first 95 target, and implement targeted strategies to reach sub-populations with larger gaps in awareness such as men and young people. HIV prevention strategies, such as condom use, remain crucial, since individual behaviors were linked to awareness of HIV status. Those unaware of their status might be less likely to adopt preventive measures such as condom use, increasing the risk of ongoing HIV transmission. HIV epidemic control is not possible while substantial numbers of PLHIV are still unaware of their status.

## Data Availability

De-identified individual participant data, data use manual, supplemental data manual, questionnaire, codebook, sampling and weighting technical report, and tabulation plan are available upon request after approval of a methodologically sound proposal. The THIS 2022−2023 dataset and materials are currently available. Data are accessible online (www.nbs.go.tz) with approved credentialled login.
